# Variation in rhizosphere microbial communities and nitrogen- and phosphorus-cycling functional genes among rubber plantations in Yunnan, China

**DOI:** 10.3389/fmicb.2026.1977925

**Published:** 2026-10-30

**Authors:** Shunjun Geng, Jifen Yang, Xiaoling Shi, Qianghao Zhang, Chunxia Yang

**Affiliations:** 1 Yunnan Key Laboratory of Sustainable Utilization Research on Rubber Tree, Xishuangbanna, China; 2 Yunnan Institute of Tropical Crops, Xishuangbanna, China

**Keywords:** functional gene, *Hevea brasiliensis*, metagenomics, nitrogen cycle, phosphorus cycle, rhizosphere microorganism

## Abstract

**Introduction:**

Rhizosphere microorganisms are important participants in soil nutrient transformation. However, variation in rhizosphere microbial community composition and nitrogen (N)- and phosphorus (P)-cycling functional gene profiles among rubber plantations in Yunnan Province remains unclear.

**Methods:**

Metagenomic sequencing was used to compare rhizosphere microbial community composition and N- and P-cycling functional gene profiles among the rubber plantations located in five major natural rubber-producing areas in Yunnan Province, China: Gengma (MD), Ximeng (XM), Mengla (ML), Jinghong (JH), and Jiangcheng (JC).

**Results:**

Bacterial and fungal community composition and N- and P-cycling functional gene profiles differed significantly among the five rubber plantations. JC exhibited higher fungal diversity and richness, but its bacterial diversity was significantly lower than that in MD. The bacterial communities were dominated by Pseudomonadota, Acidobacteriota, and Actinomycetota, and the relative abundance of Acidobacteria was significantly enriched in JC compared to JH. Furthermore, Mucoromycota, Ascomycota, and Basidiomycota were the dominant phyla in the fungal community. At the functional level, compared to JC, key genes associated with N cycling were significantly enriched in ML, including N assimilation (*glnA*, *gltB*, *gltD*), dissimilatory nitrate reduction to ammonium (*narG*, *narH*, *nirB*), nitrification (*nxrA*, *nxrB*), and denitrification (*napA*, *napB*). Furthermore, the *nosZ* gene was also significantly enriched in MD compared to JC. Regarding the P cycle, compared to JC, genes associated with high-affinity phosphate transporters (*pstA*, *pstB*, *pstC*) and polyphosphate degradation (*ndk*) were significantly enriched in MD, whereas the organic phosphorus mineralization-related gene *phnX* exhibited the opposite trend. Meanwhile, compared to MD, the polyphosphate degradation-related gene *ppk2* was significantly enriched in ML, whereas the inorganic phosphorus solubilization-related gene *pqqC* exhibited the opposite pattern. Hierarchical partitioning identified soil pH, total phosphorus (TP), and total potassium (TK) as the primary factors significantly associated with variations in bacterial community composition and P-cycling functional gene profiles.

**Conclusion:**

In summary, the composition of rhizosphere bacterial and fungal communities, as well as the functional gene profiles related to N and P cycling, differed significantly among the investigated rubber plantations. These variations were closely associated with soil physicochemical factors. These findings may provide a microbiological basis for further developing site-specific nutrient management strategies in rubber plantations.

## 1 Introduction

Soil microorganisms are integral components of biogeochemical cycles and play pivotal roles in ecosystem functioning (Fierer, 2017; Saleem et al., 2019; Van Der Heijden et al., 2007). The rhizosphere, as a critical interface for plant–microbe–soil interactions, generally differs from bulk soil in nutrient availability and microenvironmental conditions (Dennis et al., 2010; Ling et al., 2022; Reinhold-Hurek et al., 2015). Within the rhizosphere microenvironment, root exudates—including organic acids, sugars, amino acids, and signaling molecules—alter the local carbon supply, pH, and nutrient availability. Furthermore, they exert chemotactic, stimulatory, or inhibitory effects on various microbial taxa (Pascale et al., 2020; Zhang et al., 2014). These processes collectively shape the assembly and potential ecological functions of the rhizosphere microbial community (Ling et al., 2022; Nuccio et al., 2020). Rhizosphere microbes drive organic matter decomposition, N transformation, and P mineralization and solubilization, thereby influencing plant nutrient acquisition, stress resilience, and overall soil fertility (Mendes et al., 2013; Philippot et al., 2013; Trivedi et al., 2020). Therefore, deciphering the composition, functional potential, and environmental drivers of rhizosphere microbial communities is crucial for understanding soil fertility maintenance and agroecosystem sustainability.

*Hevea brasiliensis* is the primary source of natural rubber, an essential industrial material, and is cultivated mainly in tropical regions. Rubber plantation soils are typically characterized by strong acidity, a high degree of weathering, and limited nutrient retention capacity. In such soils, iron and aluminum oxides can enhance phosphorus adsorption and fixation, thereby limiting phosphorus availability (Barrow et al., 2020; Penn and Camberato, 2019). Meanwhile, nitrogen transformation is likely co-regulated by soil acidity, carbon supply, moisture status, and nitrogen fertilizer inputs (Moreau et al., 2019; Senbayram et al., 2019). Long-term monoculture and intensive management typically rely on nitrogen and phosphorus fertilizer inputs to sustain rubber tree growth and yield. Although this approach can improve nutrient supply in the short term, excessive or inappropriate fertilization is often accompanied by low nutrient use efficiency and a series of adverse environmental consequences, including aquatic eutrophication, increased emissions of greenhouse gases (e.g., nitrous oxide), and accelerated soil acidification.(Demay et al., 2023; Galloway et al., 2008; Guo et al., 2010). Consequently, deciphering the functional potential of soil microbiomes driving N and P transformations, alongside their responses to soil nutrient status, is imperative for optimizing fertilization strategies and mitigating ecological risks in rubber agroecosystems.

While previous studies on soil microbiomes in rubber plantations have primarily focused on local-scale community composition and its relationship with soil properties (Kerfahi et al., 2016; Liu et al., 2019), differences in rhizosphere microbial composition and functional potential among the rubber plantations located in the major rubber-producing areas of Yunnan remain poorly understood. Metagenomic sequencing serves as a robust tool for simultaneously characterizing the taxonomic composition and functional potential of microbial communities. Compared with traditional amplicon sequencing, metagenomics provides a more comprehensive elucidation of the genetic potential associated with microbially mediated ecological processes, such as N and P cycling, thereby advancing research from the description of community structure to the assessment of functional potential (Thomas et al., 2012). Accordingly, we collected rhizosphere soil samples from rubber plantations located in five major natural rubber-producing areas in Yunnan Province and subjected them to metagenomic sequencing. Our specific objectives were to: (1) compare the composition and diversity of rhizosphere microbial communities among the rubber plantations; (2) elucidate the differences in the abundance of key functional genes associated with N and P cycling among them; and (3) examine the coupled relationships among microbial taxa, functional gene assemblages, and key environmental variables. This study aims to deepen our understanding of variation and environmental associations within rhizosphere microecosystems across different rubber plantations, while providing foundational data to support future validation of microbially mediated nitrogen and phosphorus transformation processes, precision fertilization, and targeted rhizosphere microbiome management.

## 2 Materials and methods

### 2.1 Study area and sample collection

In this study, rubber tree rhizosphere soil samples were collected from one representative state-owned rubber enterprise plantation base in each of the five major rubber-producing regions of Yunnan Province, namely Gengma (MD), Ximeng (XM), Mengla (ML), Jinghong (JH), and Jiangcheng (JC) (Figure 1). The selected state-owned rubber enterprise plantation bases largely encompass the rubber-growing areas within their respective regions.

**Figure 1:**
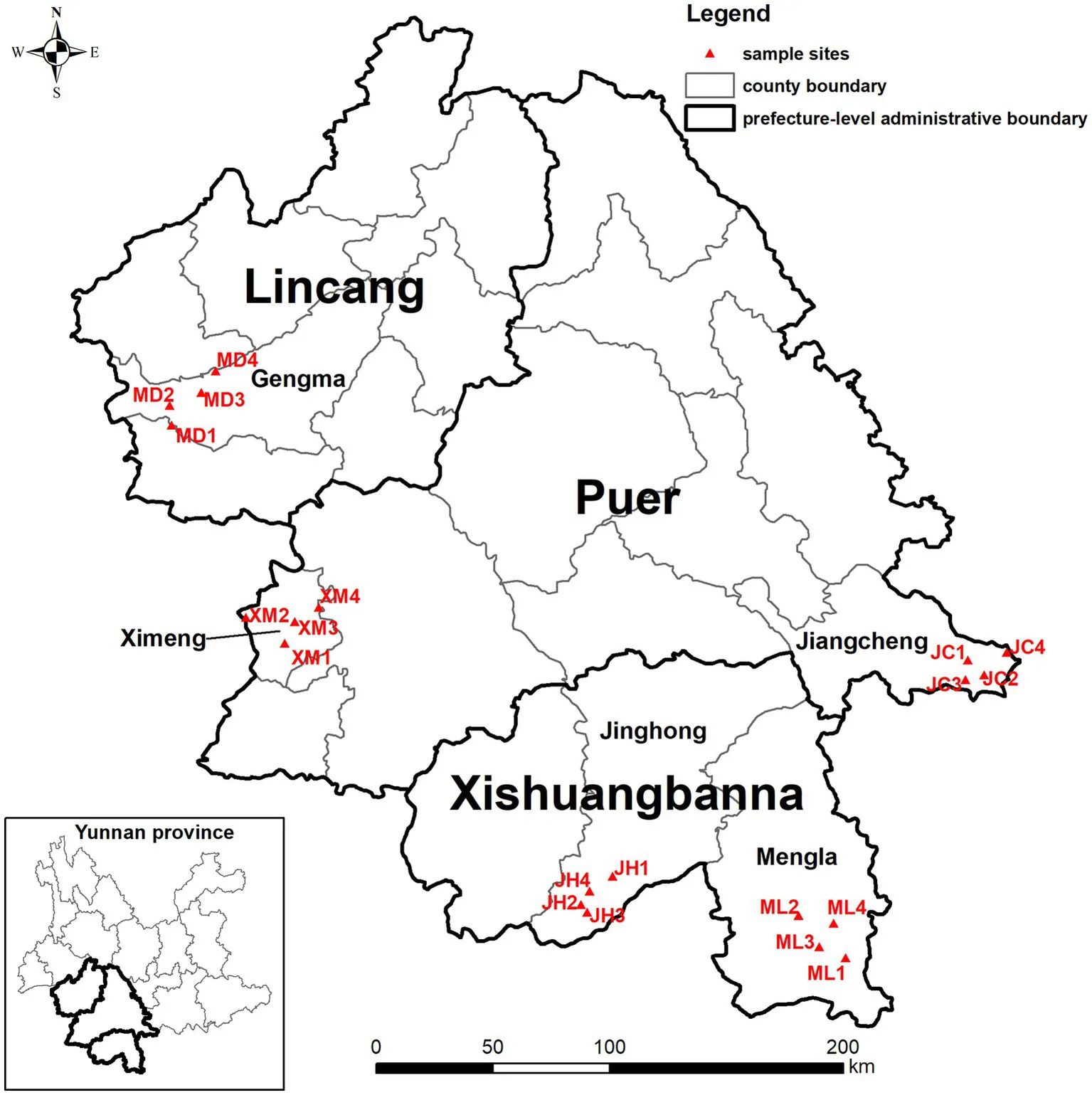
Geospatial distribution characteristics of the five sampled rubber plantations (Gengma, Ximeng, Jinghong, Mengla, and Jiangcheng, Yunnan Province) in this study.

In August 2025, four spatially separated plots (100 m × 100 m) were established within each selected plantation site, resulting in a total of 20 plot-level rhizosphere soil samples (five plantation sites × four plots per site). The four plots within each plantation site represented within-site spatial replicates and were intended to characterize within-site spatial variability; they did not represent four independent plantation-level replicates within each geographic area. Plots within each sampling location were selected based on comparable stand age (25–30 years), topography, and plantation management practices.

Within each plot, surface rhizosphere soil subsamples (0–15 cm depth) were collected using a five-point sampling method and thoroughly homogenized to form a single biological replicate. Each sample was then split into two subsamples. One subsample was passed through a 2 mm sieve, quickly frozen, and stored at −80 °C for future DNA extraction. The remaining subsample was air-dried and sieved for soil physicochemical analysis.

### 2.2 Soil metagenomic sequencing

Total genomic DNA was isolated from 0.5 g of each soil specimen utilizing the FastDNA Spin Kit for Soil (MP Biomedicals, Solon, OH, USA) in strict accordance with the manufacturer’s protocol. The DNA yield was determined via a TBS-380 fluorometer (Turner Biosystems, Sunnyvale, CA, USA), and its integrity was verified through 1% (w/v) agarose gel electrophoresis. Subsequently, the purified DNA extracts were sent to Majorbio Bio-Pharm Technology Co., Ltd. (Shanghai, China) for undergo shotgun metagenomic sequencing on the Illumina NovaSeq platform (PE150).

Raw sequencing data underwent quality control and preprocessing with fastp (v0.23.0), which involved adapter removal and the exclusion of low-quality sequences. Specifically, reads falling below 50 bp in length post-trimming or exhibiting a mean Phred quality score under 20 were eliminated. The resulting high-quality reads from all samples were then subjected to *de novo* co-assembly via MEGAHIT (v1.1.2), retaining only contigs ≥300 bp for downstream applications. Open reading frames (ORFs) were subsequently predicted from these assembled contigs employing both Prodigal (v2.6.3) (Hyatt et al., 2010) and MetaGene (Noguchi et al., 2006). Only predicted genes with nucleotide sequences of at least 100 bp were retained and subsequently translated into corresponding amino acid sequences utilizing EMBOSS (v6.6.0).

To generate a non-redundant gene catalog, the predicted gene sequences were clustered using CD-HIT (v4.6.1) under stringent criteria of 90% sequence identity and 90% coverage, with the longest sequence from each resulting cluster designated as the representative. Gene abundance estimation was performed by mapping the high-quality reads from individual samples back to this non-redundant catalog using SOAPaligner (v2.21). The alignment parameters permitted a maximum of five mismatches and enforced a minimum sequence identity of 95%.

Taxonomic classification was achieved by aligning the representative sequences against the NCBI NR database utilizing DIAMOND (v0.8.35), applying an E-value threshold of ≤1 × 10^−5^. Functional annotation was concurrently conducted by querying the Kyoto Encyclopedia of Genes and Genomes (KEGG) database (http://www.genome.jp/kegg/) via BLASTP, maintaining the same E-value cutoff (≤1 × 10^−5^). Genes implicated in N and P metabolic pathways were delineated according to their assigned KEGG Orthology (KO) identifiers. The relative abundances of these cycling-associated genes were standardized using the reads per kilobase per million mapped reads (RPKM) metric, thereby facilitating subsequent evaluations of functional profiles, differential abundance testing, and correlation analyses.

### 2.3 Determination of soil physicochemical properties

Soil pH was measured using a pH meter in a soil-to-water suspension prepared at a 1:2.5 (w/v) ratio (Bates, 1961). Soil organic matter (SOM) was quantified using the potassium dichromate external-heating method (Zhu et al., 2019), whereas total nitrogen (TN) was determined by the Kjeldahl method (Bremner, 1960). For total phosphorus (TP) analysis, soil samples were digested with concentrated H₂SO₄ and HClO₄, after which phosphorus concentrations in the resulting digests were quantified using a continuous flow analyzer (AA3, SEAL, Germany). Alkali-hydrolyzable nitrogen (AN) was determined using the alkaline hydrolysis diffusion method (Cornfield, 1960). Available phosphorus (AP) was extracted with an NH₄F solution and subsequently quantified using a continuous flow analyzer (AA3, SEAL, Germany). Available potassium (AK) was extracted with 1 mol L^−1^ ammonium acetate and measured by atomic absorption spectrophotometry (Amorim et al., 2021). Total potassium (TK) was determined following sodium hydroxide alkali fusion, with potassium concentrations subsequently measured by atomic absorption spectrophotometry (Mochen et al., 2025).

### 2.4 Statistical analyses

Principal coordinates analysis (PCoA) and mapping were performed using R (v3.3.1). Analysis of similarities (ANOSIM) and permutational multivariate analysis of variance (PERMANOVA) based on Bray–Curtis dissimilarities were conducted using the *vegan* package (v2.4.3). The Shannon and Chao1 diversity indices were calculated to assess microbial diversity using Mothur (v1.30.2; https://mothur.org/wiki/calculators/) and R (v3.3.1). Statistical analyses were performed using the Kruskal–Wallis nonparametric test followed by *post hoc* pairwise comparisons with *p*-values adjusted using the Benjamini–Hochberg false discovery rate (FDR) correction. Differences with adjusted *p* < 0.05 were considered statistically significant.

Mantel test was performed with the *vegan* package in R software (v2.6-8), and the correlations between variables were visualized using ChiPlot (https://www.chiplot.online). Redundancy analysis (RDA) was conducted to assess the effects of measured soil properties on bacterial and fungal community composition and on the abundance profiles of N- and P-cycling functional genes. Visualization was performed using *CANOCO* software (v5.0). The significance of the overall RDA models was evaluated by permutation tests (999 permutations), and adjusted *R*^2^ was used to quantify the variation in each biological profile explained by the measured soil properties. The relative contribution of each soil variable was quantified using hierarchical partitioning via the “rdacca.hp” package (Lai et al., 2022). Before analysis, all environmental factors except pH were Z-score standardized, whereas the abundances of microbial taxa and functional genes were subjected to Hellinger transformation.

## 3 Results

### 3.1 Variations in soil physicochemical properties

Several soil physicochemical properties differed significantly among the rubber plantations, whereas soil pH and AP content showed no significant differences among them (Table 1). In MD, the contents of SOM, TP, and AN were significantly higher than those in ML and JC (*p* < 0.05). These variables did not differ significantly among MD, XM, and JH. MD had the lowest TK content (*p* < 0.05), which was significantly lower than that in XM, ML, JH, and JC (*p* < 0.05). In XM, the content of TN was significantly higher than that in ML and JC (*p* < 0.05), but it did not differ significantly from that in MD and JH. Furthermore, the TK content in XM was significantly higher than that in all other rubber plantations (*p* < 0.05). Additionally, the AK content in XM was significantly higher than that in ML (*p* < 0.05), whereas no significant differences were observed among XM, MD, JH, and JC. Overall, most soil properties showed higher values in MD and XM.

**Table 1:** Soil physicochemical properties among the rubber plantations (mean ± SE).

State	MD	XM	ML	JH	JC
pH	4.82 ± 0.47a	4.62 ± 0.10a	4.52 ± 0.27a	4.65 ± 0.21a	4.71 ± 0.08a
SOM (g/kg)	52.89 ± 11.22a	51.16 ± 8.01a	21.46 ± 7.86c	44.55 ± 12.79ab	34.17 ± 9.01bc
AN (mg/kg)	293.47 ± 60.04a	283.23 ± 50.02ab	132.64 ± 39.42c	246.98 ± 48.68ab	208.08 ± 36.64b
TN (g/kg)	2.44 ± 0.60ab	2.94 ± 0.59a	1.23 ± 0.48c	2.62 ± 0.66ab	1.88 ± 0.40bc
AP (mg/kg)	6.54 ± 1.52a	5.84 ± 0.89a	7.42 ± 2.45a	5.85 ± 1.34a	6.45 ± 0.98a
TP (g/kg)	0.63 ± 0.25a	0.45 ± 0.08ab	0.17 ± 0.06c	0.53 ± 0.13a	0.30 ± 0.14bc
AK (mg/kg)	58.80 ± 19.55ab	88.76 ± 23.13a	29.70 ± 8.04b	60.53 ± 19.75a	63.27 ± 22.88a
TK (g/kg)	3.65 ± 0.45d	19.52 ± 3.46a	10.00 ± 1.48c	12.29 ± 2.32bc	15.19 ± 3.62b

Different letters in the same column indicate significant differences among the rubber plantations at *p* < 0.05. MD: Gengma; XM: Ximeng; ML: Mengla; JH: Jinghong; JC: Jiangcheng. SOM, soil organic matter; AN, alkali-hydrolyzable nitrogen; TN, total nitrogen; AP, available phosphorus; TP, total phosphorus; AK, available potassium; TK, total potassium.

### 3.2 Soil microbial community composition among the rubber plantations

PCoA revealed separation in both bacterial and fungal community composition among the rubber plantations. For bacterial communities, PC1 and PC2 explained 37.65% and 22.08% of the total variation, respectively, accounting for 59.73% cumulatively (Figure 2C). For fungal communities, PC1 and PC2 explained 39.70% and 21.28% of the total variation, respectively, with a cumulative contribution of 60.98% (Figure 3C). ANOSIM and PERMANOVA further showed significant differences in rhizosphere bacterial (ANOSIM: *R* = 0.373, *p* = 0.001; PERMANOVA: *R*^2^ = 0.362, *p* = 0.012) (Figure 2C) and fungal community composition (ANOSIM: *R* = 0.216, *p* = 0.012; PERMANOVA: *R*^2^ = 0.327, *p* = 0.023) among different rubber plantations (Figure 3C).

**Figure 2:**
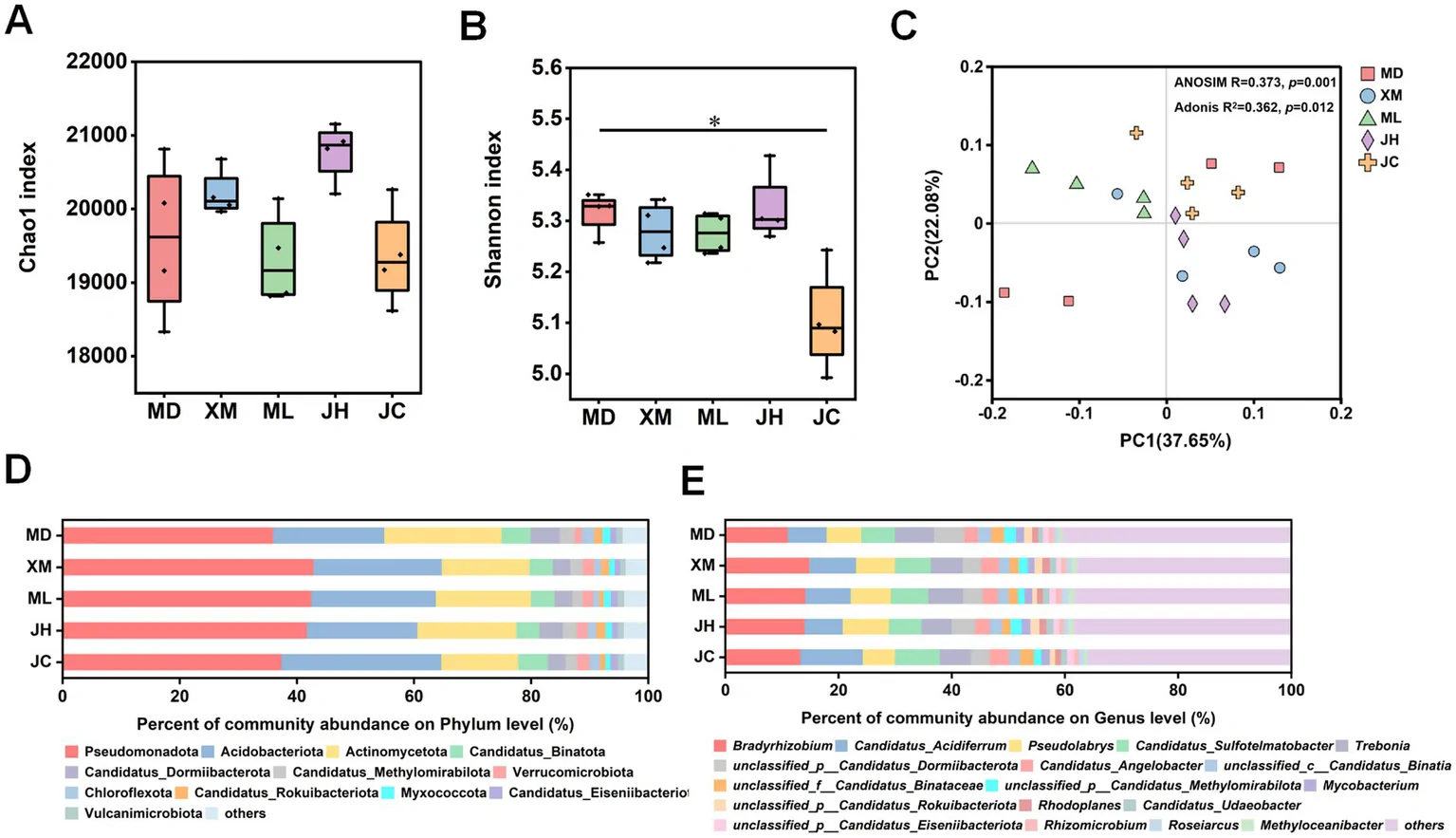
Diversity, structure, and taxonomic composition of rubber tree rhizosphere bacterial communities among the rubber plantations. **(A, B)** Chao1 and Shannon indices across different rubber plantations. **(C)** PCoA of bacterial communities at the genus level. **(D)** Bacterial community composition at the phylum level. **(E)** Bacterial community composition at the genus level. (Phyla and genera with average relative abundance (RA) < 1% were merged and indicated as “Others.”) MD: Gengma; XM: Ximeng; ML: Mengla; JH: Jinghong; JC: Jiangcheng. Significance level: ^*^p < 0.05.

**Figure 3:**
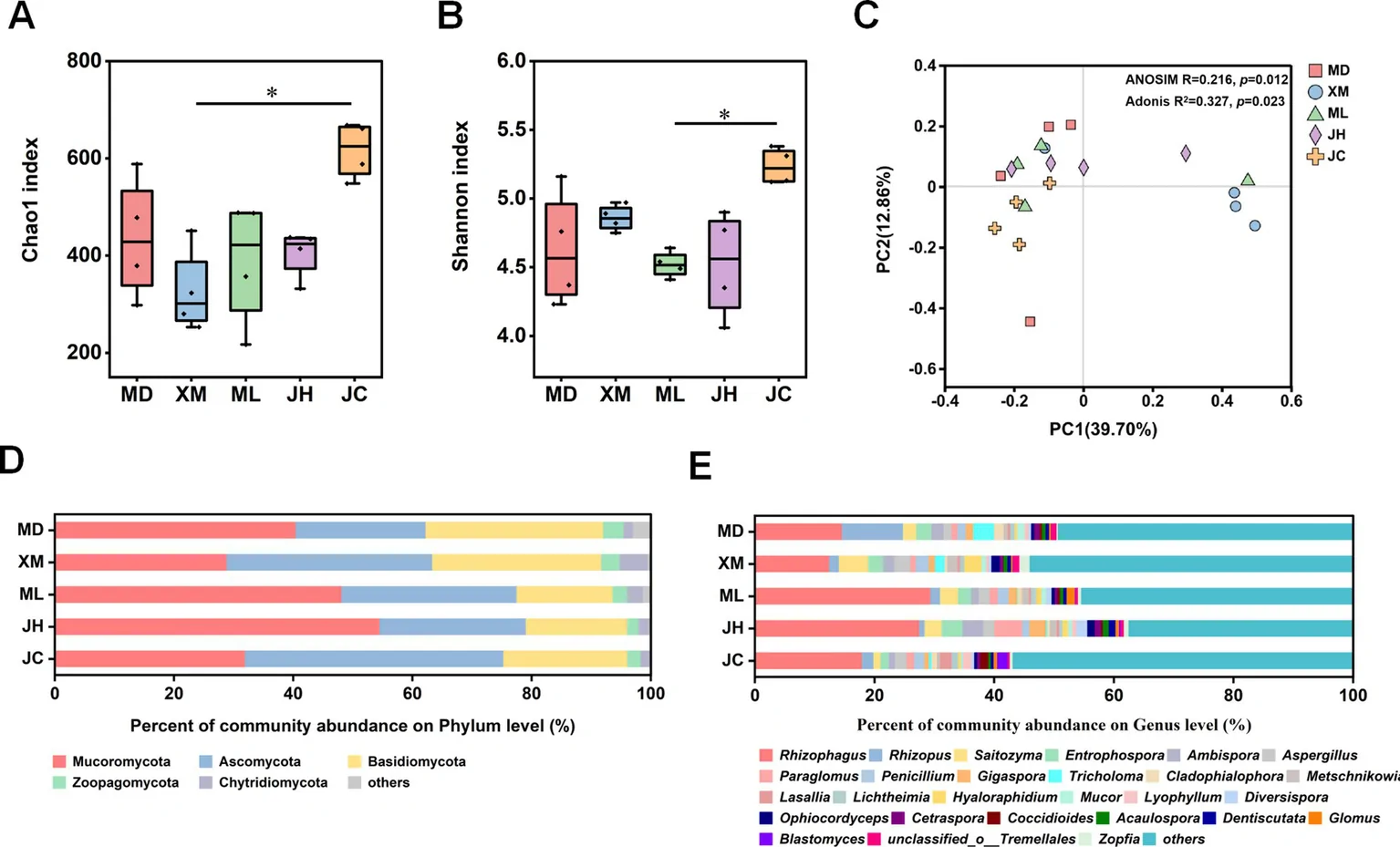
Diversity, structure, and taxonomic composition of rubber tree rhizosphere fungal communities among the rubber plantations. **(A, B)** Chao1 and Shannon indices across different rubber plantations. **(C)** PCoA of fungal communities at the genus level. **(D)** Fungal community composition at the phylum level. **(E)** Fungal community composition at the genus level. (Phyla and genera with average relative abundance (RA) < 1% were merged and indicated as “Others.”) MD: Gengma; XM: Ximeng; ML: Mengla; JH: Jinghong; JC: Jiangcheng. Asterisks indicate statistical significance: * *p* < 0.05.

#### 3.2.1 Soil bacterial community composition among the rubber plantations

The soil bacterial communities differed significantly among the rubber plantations. While the bacterial Shannon index, as estimated by the number of observed operational taxonomic units (genus level), was significantly higher in MD compared to JC (*p* < 0.05; Figure 2B), no significant differences in the overall bacterial Chao1 index were detected among the rubber plantations (Figure 2A).

At the phylum level, the bacterial communities were consistently dominated by Pseudomonadota (35.47–42.52%), Acidobacteriota (18.79–27.19%), and Actinomycetota (13.40–19.52%) among the rubber plantations (Figure 2D). However, the relative abundances of specific phyla differed among the rubber plantations. Notably, the relative abundance of Acidobacteriota in JC was significantly higher than that in JH (*p* < 0.05; Supplementary Figure S1A).

At the genus level, *Bradyrhizobium* (11.07–14.88%), *Candidatus Acidiferrum* (6.75–11.06%), and *Pseudolabrys* (5.70–8.16%) were the predominant taxa (Figure 2E). The relative abundance of *Candidatus Acidiferrum* in JC was significantly higher than that in JH (*p* < 0.05; Supplementary Figure S1B).

#### 3.2.2 Soil fungal community composition among the rubber plantations

The soil fungal communities differed significantly among the rubber plantations. The fungal Shannon index, as estimated by the number of observed operational taxonomic units (genus level), was significantly higher in JC compared to ML (*p* < 0.05; Figure 3B). Additionally, the fungal Chao1 index was significantly higher in JC compared to XM (*p* < 0.05; Figure 3A).

At the phylum level, the fungal communities were consistently dominated by Mucoromycota (28.88–54.52%), Ascomycota (21.80–43.38%), and Basidiomycota (16.10–29.76%) among the rubber plantations (Figure 3D). Notably, the relative abundances of the dominant phyla showed no significant differences among the rubber plantations (Supplementary Figure S2A).

At the genus level, the relative abundances of specific genera differed among the rubber plantations. The relative abundances of *Lyophyllum* and *Lasallia* in JC were significantly higher than those in ML, whereas the relative abundances of *Coccidioides* and *Blastomyces* in JC were significantly higher than those in XM. In addition, the relative abundance of *Zopfia* in XM was significantly higher than that in MD (*p* < 0.05; Supplementary Figure S2B).

### 3.3 Microbial functional gene composition among the rubber plantations

PCoA revealed distinct differentiation in microbial functional gene composition among the rubber plantations. The first two ordination axes explained 70.09% and 58.12% of the total variation in N- and P-cycling gene composition, respectively (Figures 4A,B). ANOSIM indicated significant differences in N- and P-cycling functional gene composition among the rubber plantations (*R* = 0.42 and 0.4001, respectively; all *p* ≤ 0.004) (Figures 4A,B).

**Figure 4:**
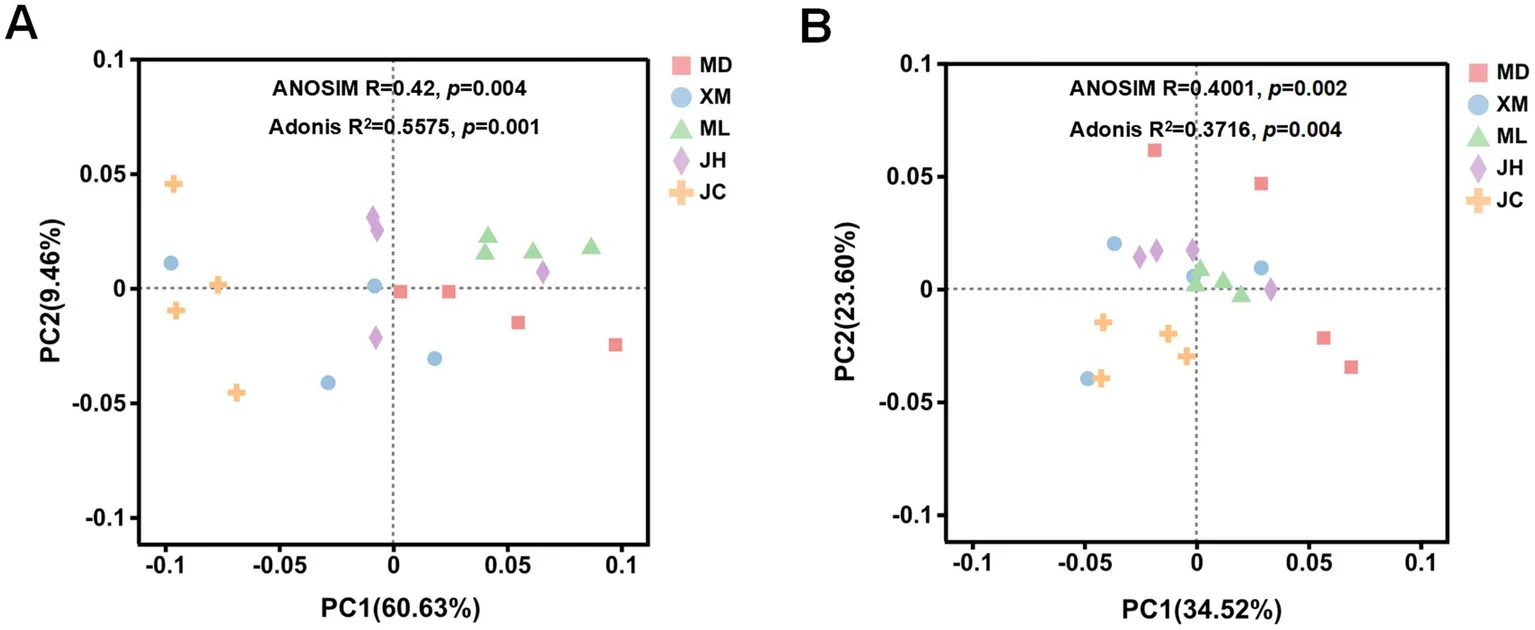
Principal coordinates analysis (PCoA) of nitrogen- **(A)** and phosphorus-cycling **(B)** functional genes based on the Bray–Curtis dissimilarity. MD: Gengma; XM: Ximeng; ML: Mengla; JH: Jinghong; JC: Jiangcheng. Percentages on axes indicate the proportion of total variance explained by each principal coordinate.

PERMANOVA further showed that plantation identity explained 55.75 and 37.16% of the variation in N- and P-cycling gene composition, respectively (all *p* ≤ 0.004) (Figures 4A,B), indicating significant differences in the functional gene composition associated with soil microbial N and P cycling among the rubber plantations.

#### 3.3.1 Nitrogen cycle functional genes among the rubber plantations

This study identified 37 genes associated with the nitrogen cycle (Supplementary Figure S3). Differential abundance analysis of N-cycling pathways revealed significant variation in several key genetic potentials among the rubber plantations. Specifically, genes associated with nitrogen assimilation, dissimilatory nitrate reduction to ammonium (DNRA), and nitrification were significantly enriched in ML compared to JC (*p* < 0.05; Figure 5B).

**Figure 5:**
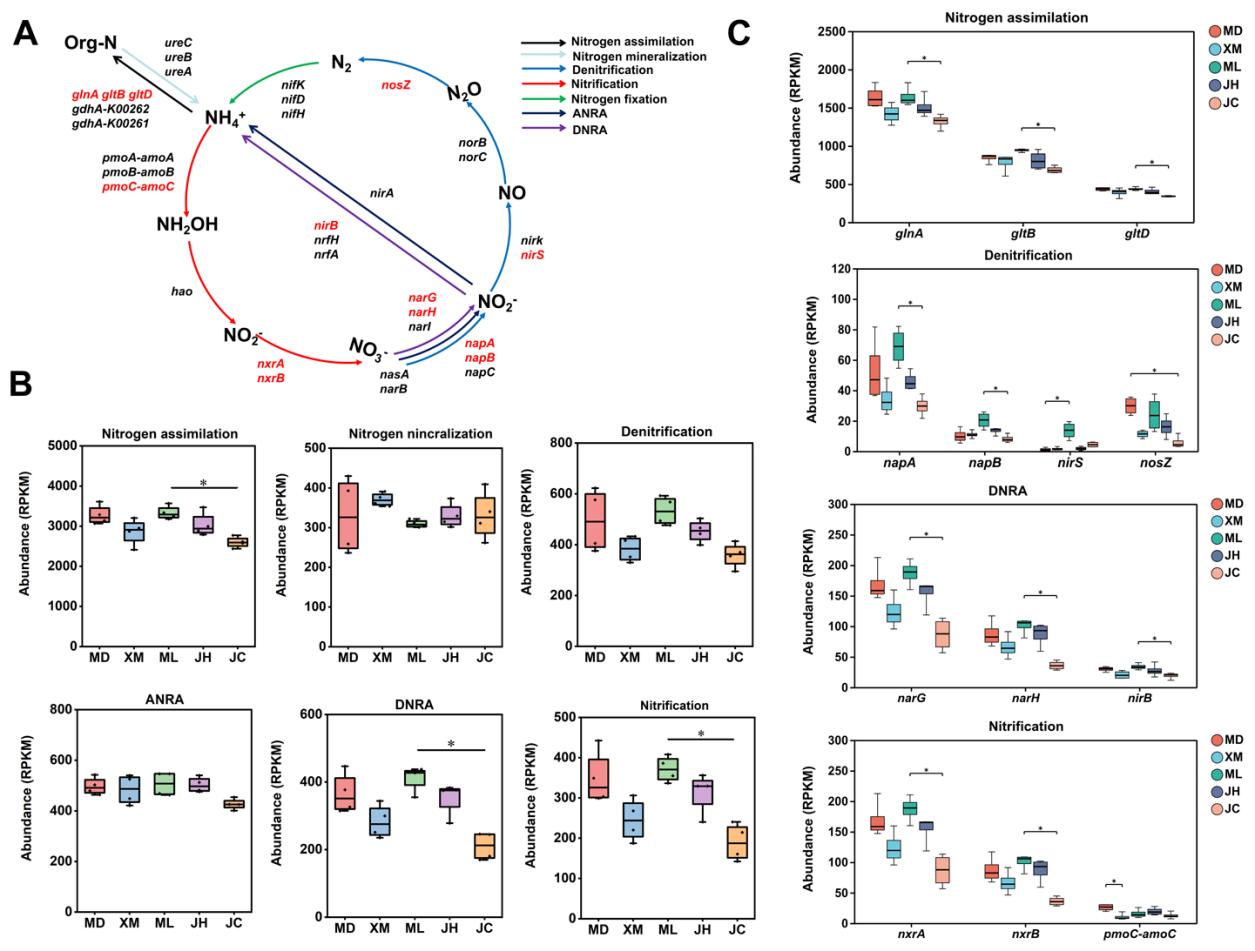
Variation in the relative abundance of nitrogen-cycling genes across different rubber plantations at the **(B)** functional level and **(C)** gene level. **(A)** Schematic diagram of the nitrogen-cycling processes. Genes that are significantly different in abundance across different rubber plantations (*p* < 0.05) are labeled in red. ANRA, Assimilatory nitrogen reduction, DNRA: Dissimilatory nitrogen reduction. MD: Gengma; XM: Ximeng; ML: Mengla; JH: Jinghong; JC: Jiangcheng. Asterisks indicate statistical significance: ^*^p < 0.05.

At the individual gene level, ML also exhibited significantly higher abundances of genes encoding key enzymes involved in the aforementioned pathways compared to JC (*p* < 0.05; Figure 5C). These included genes involved in nitrogen assimilation (*glnA*, *gltB*, *gltD*), DNRA (*narG*, *narH*, *nirB*), and nitrification (*nxrA*, *nxrB*). Among the genes involved in the denitrification pathway, *napA* and *napB* were enriched in ML compared to JC (*p* < 0.05), while *nosZ* was enriched in MD compared to JC (*p* < 0.05). Additionally, ML exhibited a significantly higher abundance of *nirS* compared to MD (*p* < 0.05).

#### 3.3.2 Phosphorus cycle functional genes among the rubber plantations

This study identified 51 genes associated with the phosphorus cycle (Supplementary Figure S4).

Differential abundance analysis of P-cycling pathways revealed significant variation in several key genetic potentials among the rubber plantations (Figure 6A). Specifically, genes associated with phosphorus transport were significantly enriched in MD compared to JC (*p* < 0.05), while genes associated with polyphosphate degradation were significantly enriched in ML compared to JC (*p* < 0.05).

**Figure 6:**
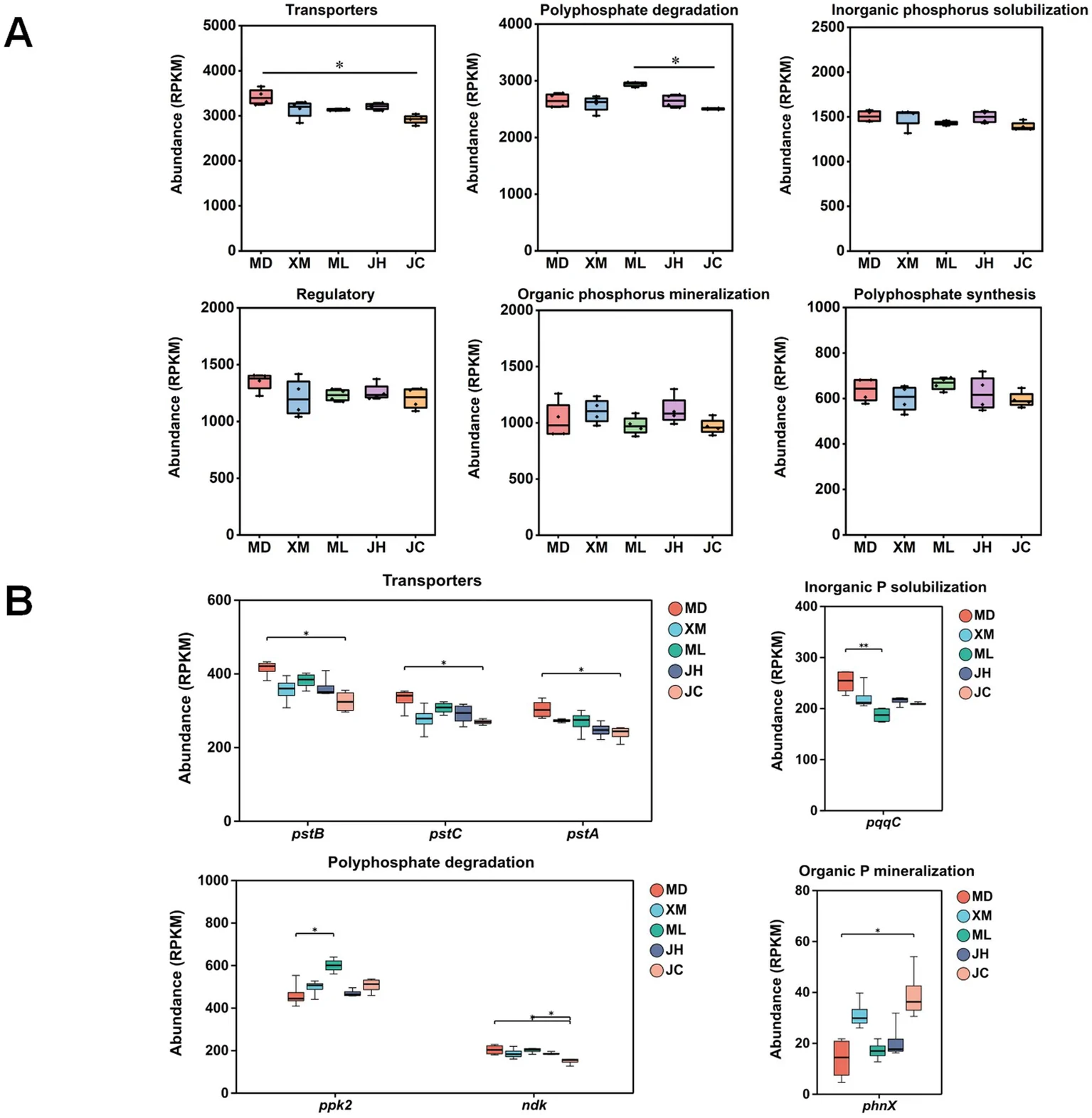
Variation in the relative abundance of phosphorus-cycling genes across different rubber plantations at the **(A)** functional level and **(B)** gene level. MD: Gengma; XM: Ximeng; ML: Mengla; JH: Jinghong; JC: Jiangcheng. Asterisks indicate statistical significance: ^*^p < 0.05, ^**^p < 0.01.

At the individual gene level, the abundances of genes encoding phosphate transport system substrate-binding protein (*pstA*, *pstB*, and *pstC*) were significantly higher in MD compared to JC (*p* < 0.05), whereas the opposite pattern was observed for the gene encoding phosphonoacetaldehyde hydrolase (*phnX*). The abundances of genes encoding pyrroloquinoline quinone (PQQ) biosynthesis (*pqqC*) were significantly higher in MD compared to ML (*p* < 0.05), whereas the opposite pattern was observed for the gene encoding polyphosphate kinase (*ppk2*). The abundances of genes encoding nucleoside-diphosphate kinase (*ndk*) were significantly higher in MD and ML compared to JC (*p* < 0.05) (Figure 6B).

### 3.4 Relationships among soil microbial communities, functional genes, and soil environmental factors

Mantel analysis showed that bacterial community composition and the abundance profiles of functional genes related to P cycling were mainly influenced by pH, TP, and TK (Figures 7A,C; Supplementary Tables S1 and S2). The fungal community composition showed a weak but significant correlation with AK (*r* = 0.1990, *p* < 0.01; Supplementary Table S1). Among the N-cycling profiles, only the gene abundance profiles related to nitrogen mineralization and denitrification were mainly influenced by SOM and AN (Figure 7B; Supplementary Table S2).

**Figure 7:**
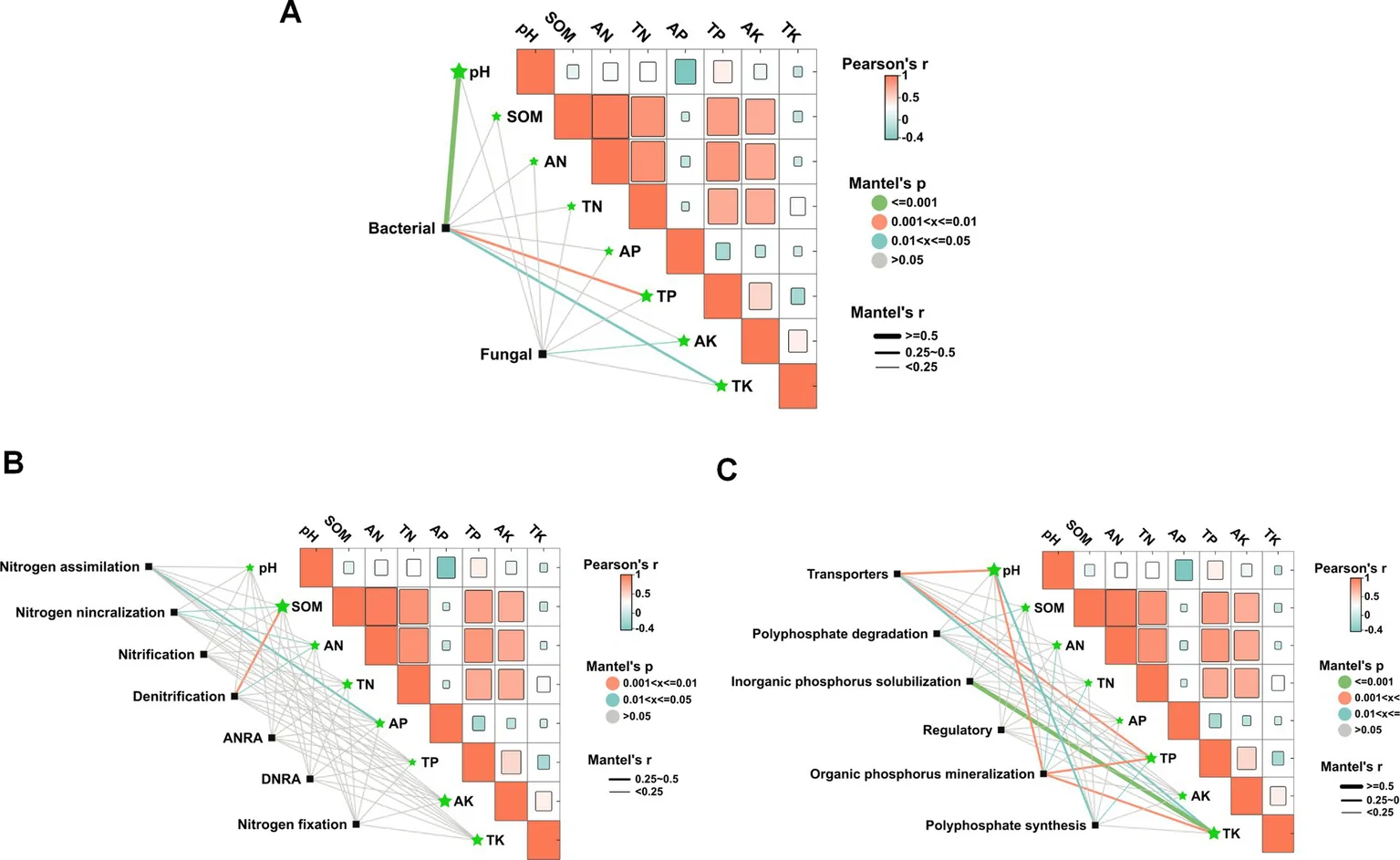
Mantel tests for the correlations between soil properties and the composition of bacterial and fungal communities **(A)**, and nitrogen- **(B)** and phosphorus-cycling **(C)** gene profiles. The color gradient represents the Pearson correlation coefficient. The width of the edge reflects Mantel’s *r* statistic, indicating the strength of distance correlation, while the edge color denotes the level of statistical significance. The size of each green triangle represents the sum of Mantel’s *r* values between the corresponding soil properties and the microbial communities **(A)** or functional gene profiles **(B, C)**. ANRA, Assimilatory nitrogen reduction, DNRA, Dissimilatory nitrogen reduction. SOM, Soil organic matter; AN, Alkali-hydrolyzable nitrogen; TN, Total nitrogen; AP, Available phosphorus; TP, Total phosphorus; AK, Available potassium; TK, Total potassium.

RDA models were significant for the bacterial community and N- and P-cycling functional gene profiles (*p* < 0.05; Figure 8A,C,D), but not significant for the fungal community (*p* > 0.05; Figure 8B). Overall, the measured soil properties accounted for 43.81%, 3.90%, 17.95%, and 36.49% of the variation in bacterial community composition, fungal community composition, and N- and P-cycling functional gene abundance profiles, respectively (adjusted *R*^2^) (Figures 8A–D).

**Figure 8:**
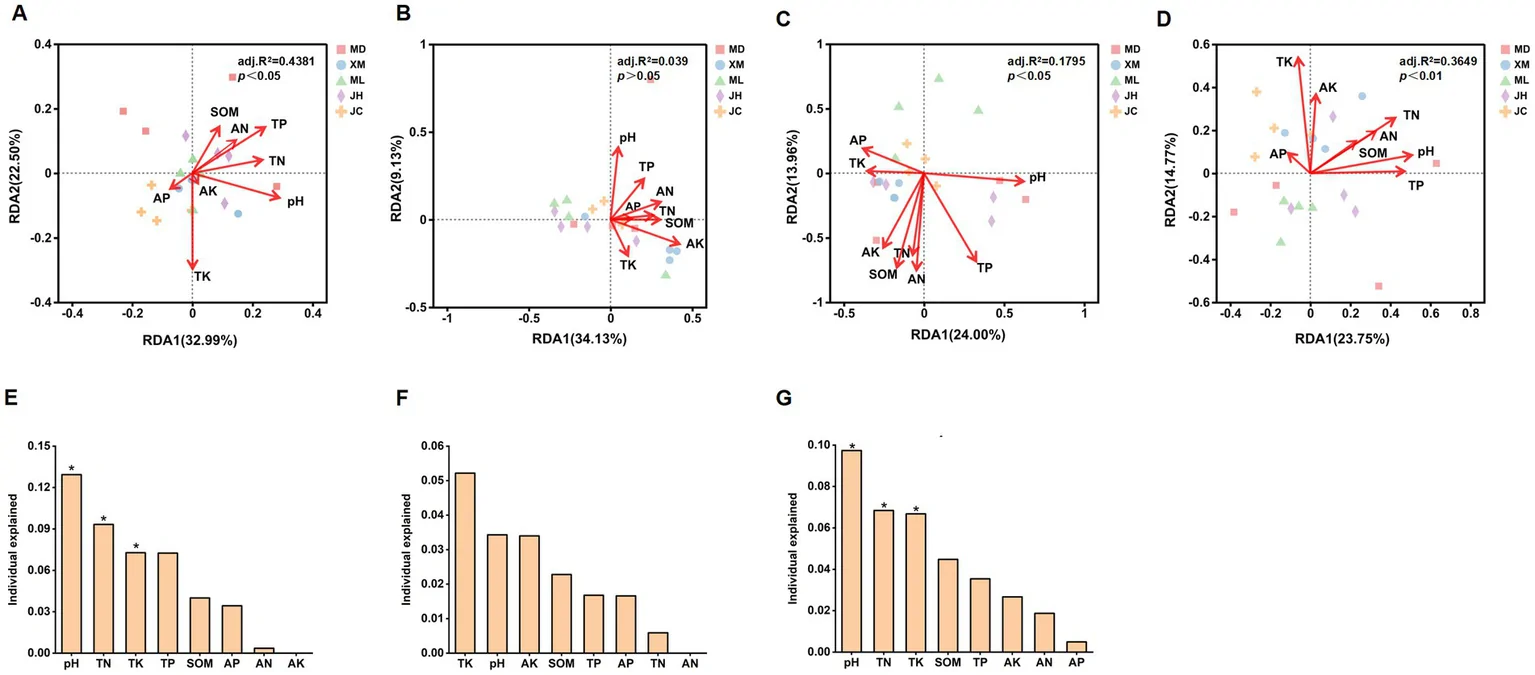
RDA of soil properties and bacterial **(A)** and fungal **(B)** communities (at the genus level), nitrogen- **(C)** and phosphorus-cycling **(D)** genes among the rubber plantations. The relative contributions of each soil parameter to these variations (except for the fungal communities) are presented **(E–G)**. SOM, Soil organic matter; AN, Alkali-hydrolyzable nitrogen; TN, Total nitrogen; AP, Available phosphorus; TP, Total phosphorus; AK, Available potassium; TK, Total potassium. Asterisks indicate statistical significance: ^*^p < 0.05, ^**^p < 0.01.

Hierarchical partitioning further indicated that soil pH, TP, and TK were the primary explanatory factors for bacterial community composition and P-cycling functional gene abundance profiles (Figures 8E,G). TK made the greatest independent contribution to variation in N-cycling functional gene profiles (Figure 8F).

## 4 Discussion

In the present study, we investigated the variation in both rhizosphere soil microbial community composition and the abundances of metagenomically annotated functional genes involved in N and P metabolism across the five different rubber plantations. Key environmental factors influencing microbial community composition and functional gene abundances were explored using hierarchical partitioning. Our results indicate that there were significant differences in both microbial community composition and the abundances of functional genes involved in N and P metabolism among the rubber plantations.

### 4.1 Variation in soil bacterial community composition and diversity among the rubber plantations

In this study, soil bacterial diversity (Shannon index) was significantly higher in MD compared to JC (*p* < 0.05; Figure 2B), whereas no significant difference in the overall bacterial richness (Chao1 index) was detected among the rubber plantations (Figure 2A). This indicates that the differences in α-diversity among the rubber plantations were mainly attributed to variations in community evenness rather than to differences in the number of taxa. Furthermore, the first two axes of the PCoA explained 59.73% of the total variance (Figure 2C). Both ANOSIM and PERMANOVA further confirmed significant differences in soil bacterial community composition among the rubber plantations (Figure 2C).

Proteobacteria, Acidobacteriota, and Actinomycetota were the dominant bacterial phyla in rhizosphere soils of rubber trees (Figure 2D), a pattern consistent with previous reports (Geng et al., 2026). JC exhibited a significantly higher relative abundance of Acidobacteriota than in JH (*p* < 0.05; Supplementary Figure S1A), along with a significant enrichment of *Candidatus*_*Acidiferrum* (*p* < 0.05; Supplementary Figure S1B). Acidobacteriota are frequently reported to be abundant in acidic or relatively nutrient-poor soils, and many members are associated with oligotrophic ecological strategies (Kalam et al., 2020; Kielak et al., 2016). This community shift may reflect the comparatively lower nutrient availability in the JC region (Table 1).

RDA showed that the measured soil properties collectively explained 43.81% of the variation in bacterial community composition (Figure 8A). Hierarchical partitioning further indicated that soil pH, TP, and TK were the primary explanatory factors for bacterial community variation (Figure 8E).

### 4.2 Variation in soil fungal community composition and diversity among the rubber plantations

Fungal α-diversity varied among the rubber plantations. Specifically, JC showed significantly higher soil fungal diversity (Shannon index) than XM and significantly higher soil fungal richness (Chao1 index) than ML (*p* < 0.05; Figures 3A,B). Compared with bacterial α-diversity, fungalα-diversity exhibited a distinct variation pattern. This discrepancy may be related to fungal mycelial networks and spatial foraging capacity reported in previous studies (Aleklett et al., 2021; Peay et al., 2016). Furthermore, the first two axes of the PCoA explained 60.98% of the total variance (Figure 3C). Both ANOSIM and PERMANOVA further confirmed significant differences in fungal community composition among the rubber plantations (Figure 3C). The relative abundances of dominant fungal phyla showed no significant differences among the rubber plantations (Supplementary Figure S2A), yet exhibited significant variation at the genus level (Supplementary Figure S2B), a pattern consistent with previous reports (Ning et al., 2025).

RDA showed that the measured soil properties collectively explained 3.90% of the variation in fungal community composition (Figure 8B). However, the overall model was not significant (*p* > 0.05; Figure 8B). Thus, the relationship between the measured soil factors and fungal community composition was not statistically supported. Future studies should incorporate broader environmental parameters and integrate null-model-based community assembly analyses and neutral community models to further quantify the relative contributions of deterministic and stochastic processes to rhizosphere soil fungal community assembly in rubber plantations.

### 4.3 Variation in microbial nitrogen transformation and retention potential among the rubber plantations

Soil microbe-mediated nitrogen transformation processes are key mechanisms that regulate nitrogen availability in ecosystems and their environmental impacts (Kuypers et al., 2018; Norton and Ouyang, 2019). ML exhibited enhanced functional potential for several key nitrogen-cycling processes—specifically nitrogen assimilation, nitrification, and DNRA—compared to JC (Figure 5B). At the individual gene level, genes involved in nitrogen assimilation (*glnA*, *gltB*, and *gltD*) were significantly enriched in ML (Figure 5C). These genes collectively encode components of the glutamine synthetase/glutamate synthase (GS/GOGAT) system, which is a central pathway for incorporating ammonium into organic nitrogen compounds (Geisseler et al., 2010). Their enrichment suggests that the ML microbial community has greater genetic potential for assimilating inorganic N into microbial biomass.

Nitrogen transformation processes, including nitrification and denitrification, are important regulators of N loss and nitrous oxide (N₂O) emissions (Isobe et al., 2020; Kuypers et al., 2018; Norton and Ouyang, 2019). Compared with JC, ML were significantly enriched in the nitrite-oxidation genes *nxrA* and *nxrB* (encoding subunits of nitrite oxidoreductase, mediatingNO₂^−^ oxidation to NO₃^−^), as well as nitrate-reduction genes *napA* and *napB* (encoding subunits of periplasmic nitrate reductase, mediating NO₃^−^ to NO₂^−^). The enrichment of *nxrA* and *nxrB* indicates an enhanced genetic potential for nitrite oxidation and may contribute to nitrate availability for downstream nitrate-reduction pathways, including DNRA and denitrification.

In the DNRA pathway, *narG* and *narH* encode subunits of the membrane-bound nitrate reductase (reducing NO₃^−^ to NO₂^−^), while *nirB* encodes an NADH-dependent nitrite reductase (reducing NO₂^−^ to NH₄^+^). The significantly higher abundances of these genes in ML suggest enhanced genetic potential for DNRA and, consequently, greater microbial retention of N as ammonium, which may partially alleviate N limitation. Collectively, these results indicate that the ML microbial community has greater overall genetic potential for N transformation and retention than that in JC.

In addition, the abundance of the *nosZ* gene, which encodes nitrous oxide reductase and catalyzes the reduction of N₂O to N₂, was significantly higher in MD than in JC (Figure 6C). This enrichment suggests that the MD microbial community has greater genetic potential for N₂O consumption, which could potentially mitigate soil N₂O emissions (Krause et al., 2017).

RDA indicated that the measured soil properties collectively accounted for 17.95% of the variation in nitrogen-cycling functional gene abundances (Figure 8C). Among these variables, soil TK made the greatest independent contribution to this variation (Figure 8F).

### 4.4 Variation in microbial phosphorus acquisition and transformation potential among the rubber plantations

Phosphorus (P) is an essential macronutrient for plants, but its limited bioavailability in soil often constrains agricultural productivity, particularly in highly weathered tropical ecosystems (Du et al., 2020; Peñuelas et al., 2026). Compared with JC, the MD microbial community exhibited greater genetic potential for P transport, as indicated by the significantly higher abundances of the high-affinity phosphate transporter genes *pstA*, *pstB*, and *pstC* (Figure 6B). In addition, the *ndk* gene, which contributes to maintaining nucleotide homeostasis and the balance of intracellular NTP pools (Bernard et al., 2000; Lu and Inouye, 1996), was significantly enriched in MD. These patterns suggest that the MD microbial community has an enhanced capacity for high-affinity phosphate uptake and energy regulation. However, the gene associated with phosphonate utilization, *phnX* (encoding phosphonoacetaldehyde hydrolase), was significantly less abundant in MD than in JC. Its relative enrichment in JC suggests that the JC microbial community possesses a greater genetic potential to mineralize recalcitrant organic P compounds, particularly phosphonates.

Furthermore, *pqqC* was significantly more abundant in MD than in ML, whereas *ppk2* showed the opposite pattern (Figure 6B). The *pqqC* gene is involved in PQQ biosynthesis, which has been associated with gluconic acid-mediated solubilization of sparingly soluble inorganic phosphates (Magnusson et al., 2004; Meyer et al., 2011). The *ppk2* gene encodes polyphosphate kinase 2, which transfers phosphate groups from polyphosphate (polyP) to nucleotides, thereby contributing to nucleotide regeneration and polyP metabolism (Nocek et al., 2008; Zhang et al., 2002). Collectively, the MD microbial community may exhibit greater genetic potential for high-affinity phosphate transport and PQQ-associated inorganic phosphate solubilization, whereas the JC microbial community may have greater genetic potential for phosphonate utilization and the ML microbial community may have greater genetic potential for polyP metabolism.

RDA showed that the measured soil properties collectively explained 36.49% of the variation in P-cycling functional gene abundances (Figure 8D). Hierarchical partitioning further indicated that soil pH, TP, and TK were the primary explanatory factors for this variation (Figure 8G).

### 4.5 Nutrient management implications of variation in microbial nutrient-cycling potential among rubber plantations

Our findings reveal differentiation in rhizosphere microbial N- and P-cycling gene profiles among the rubber plantations, establishing a microbiological basis for site-specific nutrient management in Yunnan rubber plantations. However, metagenomic functional gene abundances reflect functional potential rather than actual nutrient transformation activity, and therefore the corresponding strategies still require field validation. In ML, the enrichment of genes involved in N assimilation, nitrification, and DNRA suggests that the microbial community may possess a higher genetic potential for N turnover and nitrogen retention, which may favor sustaining soil nitrogen supply. However, an enhanced nitrification potential may also amplify the risk of nitrate leaching and N₂O emissions. Accordingly, split nitrogen applications and the use of slow- or controlled-release fertilizers could be considered to improve nitrogen-use efficiency and reduce nitrogen losses. In MD, the higher abundances of *nosZ*, high-affinity phosphate transporter genes (*pstA*, *pstB*, and *pstC*), and *pqqC* suggest that this region may possess a stronger potential for N₂O reduction, phosphate acquisition, and the solubilization of recalcitrant inorganic phosphorus. These functional traits may help improve soil phosphorus availability, thereby alleviating the impact of phosphorus limitation on nutrient uptake and growth in rubber trees. In this region, the potential for using poorly soluble phosphorus sources alongside organic amendments can be assessed by examining the levels of phosphorus available in the soil. In contrast, the higher abundance of the *phnX* gene in JC indicates that the microbial community may possess greater genetic potential for utilizing organic P compounds, such as phosphonates. This may reflect limited phosphorus availability in the region; therefore, management should focus on improving P supply through rational P supplementation and soil pH regulation.

Soil pH, TP, and TK were the principal soil factors significantly associated with the variation in P-cycling gene profiles, whereas TK accounted for the greatest independent proportion of the variation in N-cycling gene profiles. It is recommended that region-specific management be grounded in soil testing and adopt the integrated regulation of soil pH, organic material inputs, and P and K supplies, thereby improving nutrient availability, enhancing nutrient uptake by rubber trees, and minimizing nutrient losses.

## 5 Conclusion

This study systematically characterized the variation in rhizosphere microbial communities and their functional gene profiles associated with nitrogen (N) and phosphorus (P) cycling across five major natural rubber plantations in Yunnan Province. Significant differences were observed in rhizosphere bacterial and fungal community composition, as well as in N- and P-cycling functional gene profiles, among the rubber plantations. The measured soil physicochemical properties were significantly associated with variation in bacterial community composition and N- and P-cycling gene profiles (*p* < 0.05), but showed no significant explanatory effect on fungal community variation (*p* > 0.05), indicating distinct responses of bacterial and fungal communities to the measured soil environmental gradients. At the functional gene level, differences were detected in the abundances of genes associated with N assimilation, nitrite oxidation, DNRA, phosphate transport, phosphonate utilization, PQQ biosynthesis, and polyphosphate metabolism, suggesting heterogeneity in the genetic potential of rhizosphere microbial communities for N and P cycling among the rubber plantations. Soil pH, TP, and TK were the principal soil factors significantly associated with variation in bacterial community composition and P-cycling gene profiles, whereas TK accounted for the greatest independent proportion of variation in N-cycling gene profiles.

Overall, rhizosphere microbial communities in rubber plantations in Yunnan Province exhibited pronounced differentiation in taxonomic composition and N- and P-cycling genetic potential, which was closely associated with local soil physicochemical conditions. These findings provide a microbiological basis for developing region-specific nutrient management strategies in rubber plantations. However, metagenomic functional gene abundances reflect functional potential rather than actual nutrient transformation activity. Future studies should integrate functional gene transcriptional analyses, soil enzyme activity assays, stable-isotope tracing, or direct measurements of N and P transformation rates, together with cross-seasonal sampling and field management experiments, to further clarify the relationships among rhizosphere microbial functions, nutrient cycling, and plant nutrient utilization in rubber plantations.

## Data Availability

The datasets presented in this study can be found in online repositories. The names of the repository/repositories and accession number(s) can be found at: https://www.ncbi.nlm.nih.gov/, PRJNA1542919.
