# Supplementary material for: Variation in rhizosphere microbial communities and nitrogen- and phosphorus-cycling functional genes among rubber plantations in Yunnan, China
Source: Front Microbiol. 2026 Oct 30;17:1977925. doi: 10.3389/fmicb.2026.1977925 (PMC13759755; doi:10.3389/fmicb.2026.1977925)
Supplement: Supplementary file 1 [file Supplementary_file_1.docx]

**Supplementary Information**

**Supplementary Table S1** Mantel tests between soil factors and the composition of bacterial and fungal communities.

|  | **Mantel’s r** | |
| --- | --- | --- |
|  | **Bacterial** | **Fungal** |
| pH | 0.6143*** | 0.2180 |
| SOM | 0.0023 | -0.1112 |
| AN | 0.0527 | -0.0005 |
| TN | -0.0261 | -0.0189 |
| AP | 0.0513 | -0.0224 |
| TP | 0.4130** | 0.0703 |
| AK | -0.1956 | 0.1990** |
| TK | 0.2867* | 0.1469 |

SOM: soil organic matter; AN: alkali-hydrolyzable nitrogen; TN: total nitrogen; AP: available phosphorus; TP: total phosphorus; AK: available potassium; TK, total potassium. Asterisks indicate statistical significance: * *P* < 0.05, ** *P* < 0.01, *** *P* < 0.001.

**Supplementary Table S2** Mantel tests between soil factors and nitrogen- and phosphorus-cycling gene profiles.

| **N-cycling genes** | **Mantel’s r** | | | | | | |
| --- | --- | --- | --- | --- | --- | --- | --- |
|  | **nitrogen assimilation** | **Nitrogen nincralization** | **Nitrification** | **Denitrification** | **ANRA** | **DNRA** | **Nitrogen fixation** |
| pH | 0.1553 | 0.0093 | -0.0981 | 0.0480 | 0.1692 | -0.0013 | 0.0483 |
| SOM | -0.0335 | 0.194* | -0.0918 | 0.3039** | 0.0674 | -0.1054 | 0.1191 |
| AN | -0.0359 | 0.1654* | -0.0822 | 0.2142* | 0.0755 | 0.0175 | 0.0390 |
| TN | -0.0845 | 0.1424 | -0.0810 | 0.1399 | 0.0846 | 0.0104 | -0.1480 |
| AP | 0.2622* | -0.0615 | -0.1027 | 0.0174 | -0.0112 | -0.0567 | -0.1366 |
| TP | 0.1320 | 0.0265 | -0.0467 | 0.1273 | 0.0847 | 0.0837 | -0.0002 |
| AK | -0.1080 | 0.1075 | 0.2091 | 0.1567 | 0.0912 | 0.1158 | -0.0605 |
| TK | 0.1479 | 0.1103 | 0.0283 | 0.1469 | 0.0952 | 0.1003 | 0.1370 |
| **P-cycling genes** | **Mantel’s r** | | | | | | |
|  | **Transporters** | **Polyphosphate degradation** | **Inorganic phosphorus solubilization** | **Regulatory** | **Organic phosphorus mineralization** | **Polyphosphate synthesis** | |
| pH | 0.4639** | 0.1612 | 0.2078 | 0.1778 | 0.4410** | 0.3262* | |
| SOM | 0.1152 | 0.1897* | 0.0420 | -0.0064 | 0.1655 | -0.1180 | |
| AN | 0.1642 | 0.1264 | 0.1060 | -0.0297 | 0.2421* | 0.0163 | |
| TN | 0.0108 | 0.0034 | 0.0468 | -0.0482 | 0.2473* | -0.0033 | |
| AP | 0.0162 | 0.0071 | 0.0745 | -0.0117 | -0.0013 | 0.0290 | |
| TP | 0.3809** | 0.0592 | 0.0430 | -0.0212 | 0.3846** | 0.2121* | |
| AK | -0.0994 | -0.0781 | 0.1003 | -0.1728 | 0.0436 | -0.0376 | |
| TK | 0.2547* | 0.2071* | 0.5079*** | 0.1342 | 0.3118* | 0.1587 | |

SOM: soil organic matter; AN: alkali-hydrolyzable nitrogen; TN: total nitrogen; AP: available phosphorus; TP: total phosphorus; AK: available potassium; TK, total potassium. Asterisks indicate statistical significance: * *P* < 0.05, ** *P* < 0.01, *** *P* < 0.001.

**Supplementary Figure S1**

Analysis of differences in rhizosphere bacterial community composition among the rubber plantations. (A) phylum level; (B) genus level. MD: Gengma; XM: Ximeng; ML: Mengla; JH: Jinghong; JC: Jiangcheng. Asterisks indicate statistical significance: * *P* < 0.05.

**Supplementary Figure S2**

Analysis of differences in rhizosphere fungal community composition among the rubber plantations. (A) phylum level; (B) genus level. MD: Gengma; XM: Ximeng; ML: Mengla; JH: Jinghong; JC: Jiangcheng. Asterisks indicate statistical significance: * *P* < 0.05.

**Supplementary Figure S3**

Heatmap of nitrogen-cycling gene abundance. MD: Gengma; XM: Ximeng; ML: Mengla; JH: Jinghong; JC: Jiangcheng.

**Supplementary Figure S4**

Heatmap of phosphorus-cycling gene abundance. MD: Gengma; XM: Ximeng; ML: Mengla; JH: Jinghong; JC: Jiangcheng.
